# Relevance of the Materno-Fetal Interface for the Induction of Antigen-Specific Immune Tolerance

**DOI:** 10.3389/fimmu.2020.00810

**Published:** 2020-05-14

**Authors:** Angelina Mimoun, Sandrine Delignat, Ivan Peyron, Victoria Daventure, Maxime Lecerf, Jordan D. Dimitrov, Srinivas V. Kaveri, Jagadeesh Bayry, Sébastien Lacroix-Desmazes

**Affiliations:** ^1^Centre de Recherche des Cordeliers, INSERM, Sorbonne Université, Université de Paris, Paris, France; ^2^HITh, INSERM, UMR_S1176, Université Paris-Sud, Université Paris-Saclay, Le Kremlin-Bicêtre, France

**Keywords:** neonatal Fc receptor (FcRn), maternal IgG, immune system ontogeny, immune tolerance induction, hemophilia A, therapy

## Abstract

In humans, maternal IgGs are transferred to the fetus from the second trimester of pregnancy onwards. The transplacental delivery of maternal IgG is mediated by its binding to the neonatal Fc receptor (FcRn) after endocytosis by the syncytiotrophoblast. IgGs present in the maternal milk are also transferred to the newborn through the digestive epithelium upon binding to the FcRn. Importantly, the binding of IgGs to the FcRn is also responsible for the recycling of circulating IgGs that confers them with a long half-life. Maternally delivered IgG provides passive immunity to the newborn, for instance by conferring protective anti-flu or anti-pertussis toxin IgGs. It may, however, lead to the development of autoimmune manifestations when pathological autoantibodies from the mother cross the placenta and reach the circulation of the fetus. In recent years, strategies that exploit the transplacental delivery of antigen/IgG complexes or of Fc-fused proteins have been validated in mouse models of human diseases to impose antigen-specific tolerance, particularly in the case of Fc-fused factor VIII (FVIII) domains in hemophilia A mice or pre-pro-insulin (PPI) in the case of preclinical models of type 1 diabetes (T1D). The present review summarizes the mechanisms underlying the FcRn-mediated transcytosis of IgGs, the physiopathological relevance of this phenomenon, and the repercussion for drug delivery and shaping of the immune system during its ontogeny.

## Introduction

The existence of a passive transfer of immunity from the mother to the young was documented by P. Ehrlich more than a century ago and more than 50 years ago by Brambell et al. (1); it was a few years before the demonstration that passive transfer of immunity is mediated by maternal IgGs. The maternal and fetal circulations are separated by cellular barriers differently organized depending on the species [hemomonochorial in the human (2) and hemotrichorial in the mouse]. In 1964, Brambell et al. hypothesized that the transplacental delivery of maternal IgGs involves a receptor expressed by placental cells (3). A few years later, a receptor responsible for the trans-epithelial transport of IgGs across the newborn rat intestine was identified (4, 5). The surface and intracellular expression of the Fc receptor by the placenta (6) or yolk sac cells (7) and intestinal cells (3, 7–10) and its colocalization with IgGs (4, 6) suggested its involvement in the transfer of maternal IgGs. It led to the isolation from human placenta of this “IgG transporter” and its identification as the neonatal Fc receptor (FcRn) (11, 12).

In addition to the transplacental delivery of maternal IgGs, the FcRn is involved in a plethora of functions including the transfer of IgGs present in maternal milk to the newborn through the digestive epithelium, control of IgG and albumin catabolism, uptake of immune complexes by a variety of cells leading, in the case of antigen presenting cells, to the presentation of the endocytosed antigen to T lymphocytes. In the recent years, strategies that exploit the transplacental delivery of antigen/IgG complexes or of Fc-fused proteins have been validated in mouse models of human diseases to trigger antigen-specific immune tolerance. The present review summarizes the mechanisms underlying the FcRn-mediated transcytosis of IgGs, the physio-pathological relevance of this phenomenon and the potential for *in utero* drug delivery and manipulation of the immune system.

## Structure and Expression of FcRn

**Figure 1:**
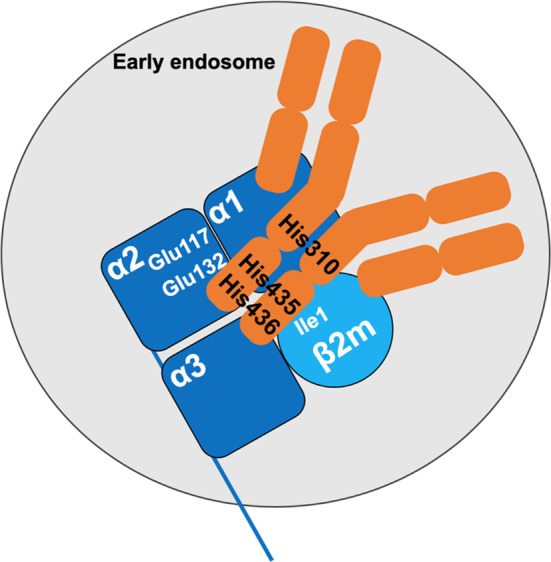
Interaction between the FcRn and IgG. The FcRn is composed of a heavy chain with three extracellular domains (α1, α2, α3, dark blue) and of the β-2 microglobulin light chain (β2 m, light blue). At acidic pH, salt bridges are formed upon interactions between the histidine residues His310, His435, and His436 of the CH2 and CH3 domains of the IgG and glutamate residues Glu117 and Glu132 of the α2 domain of the heavy chain of FcRn, and the isoleucine residue Ile1 of the β 2 m. The IgG is depicted in orange.

FcRn was first isolated from rat intestinal epithelial cells (4, 13, 14), rodent yolk sac (7), and finally from human syncytiotrophoblast cells (15, 16). FcRn is a heterodimeric molecule constituted of a 14 kDa light chain and a 45–50 kDa heavy chain (14). The heavy chain includes 3 extracellular domains (α1, α2, and α3), a transmembrane domain, that allows anchoring to cell membranes, and a short cytoplasmic domain (17) (Figure 1). The α1 and α2 domains are formed of 8 antiparallel ß-sheets overhung by 2 α-helices (18–20). The structural homology of the FcRn with the major histocompatibility complex class I (MHC-I) was confirmed by the homology between the coding sequences of the extracellular domains and transmembrane region of FcRn and MHC-I, and by crystallography (7, 9, 16, 18, 21). The heavy chain and light chain-encoding genes are highly conserved across mammalian species (22–24). Thus, human FCGRT (Fc fragment of IgG receptor and transporter) gene and mouse ortholog (*Fcgrt*) encoding FcRn present a strong sequence homology with 69 and 65% identity at the nucleotide and amino-acid levels, respectively, and low allelic polymorphism (22–24). The FCGRT and *Fcgrt* genes are located outside the HLA/H2 genes complex, on the 19q13 locus in human and on chromosome 7 in mice, respectively. The absence of the ß-microglobulin chain hampers the conformation and functionality of the FcRn (25) which was used advantageously in ß2 m^−/−^ mice to demonstrate the implication of FcRn in IgG transmission (26). Moreover, more evidence came later with the development of FcRn heavy chain KO mice (27).

During fetal life in rodents, FcRn is expressed by cells of the yolk sac (7, 22), and, to a greater extent, by epithelial cells in the jejunum and duodenum (8) where it is maintained until the time of weaning (3 weeks after birth) and mediates the transfer of IgGs contained in the colostrum or maternal milk. After weaning, FcRn expression in the digestive epithelium is highly reduced (13, 14, 28, 29). FcRn expression has also been detected in rodent skin, spleen, liver, and muscle vascular endothelial cells (30–33). Conversely, in humans, FcRn expression by intestinal epithelial cells persists during adult life (10, 34). The heavy and light chains of human FcRn are synthesized by syncytiotrophoblast cells (6, 16, 35, 36) and by arterial or vascular endothelial cells of the placenta (37, 38). Human FcRn is detected in different tissues including the liver, kidneys, lungs, heart, pancreas and mammary glands (15, 39, 40); it is expressed by hematopoietic cells (dendritic cells, monocytes, macrophages, and neutrophils, B lymphocytes) but not by T lymphocytes and natural killer (NK) cells (41–43). Differences in FcRn expression between humans and mice are explained by differences in the promoters controlling FCGRT expression (24, 44).

## Mechanisms of FcRn-Mediated IgG Transport

The dependency on pH of the interaction between IgG and the FcRn was described in different experimental settings. IgGs in maternal milk bind to intestinal FcRn at pH 6-6.5 and are released at pH 7.4 (45). The same was found for IgG binding to placental membranes (13, 46–49). While the increased binding observed at acidic pH was initially thought to rely on conformational changes in FcRn (50), it was later found that acidification allows protonation of histidine residues in the heavy chain of FcRn, thus stabilizing the FcRn molecule by fostering electrostatic interactions (17, 19). Furthermore, the CH2 and CH3 domains of the IgG heavy chain also contain three histidine residues, that are highly conserved between species (51, 52). At pH <6, His310, His435, and His436 in the mouse IgG_1_ are protonated. This allows the formation of saline bridges with glutamate 117 and 132 and an aspartate residue inside an anionic pocket of the α2 domain of the FcRn (17, 19, 21, 47, 50) as well as the Ile1 of the ß2 m (53) (Figure 1). Of note, alanine substitutions of the Ile253, His310 and His435 abrogate the binding of human IgG_1_ to the FcRn at acidic pH (54). Increase in pH above 6 leads to the loss of CH2-CH3/FcRn interaction because of the deprotonation of the histidine residues. Crystallographic investigations show that two FcRn molecules bind a single IgG through each of the Fc fragments (18). FcRn binding demonstrates a strong specificity for the IgG isotype. In humans, IgA, IgM, IgD, and IgE are not or only poorly transported through the placenta (43–47). The binding of the different IgG subclasses to FcRn also depends on variations in amino-acid sequences in the CH2 and CH3 domains leading to different affinities for FcRn (55). Indeed, human IgG1 and IgG4 are the most transferred IgG subclasses, while IgG3 which possesses an arginine rather than a histidine at position 435, presents a reduced transplacental delivery and a three-fold lower half-life than the other IgG subtypes (55–60). Interestingly, the binding affinity for the FcRn of an IgG of a given subclass is also influenced by the nature of its complementarity determining regions (CDR) and antigen-binding fragments (Fab) (61–63). Likewise, the glycosylation profile of a given IgG subclass has an impact on IgG transfer and transplacental delivery of maternal IgG by modifying the affinity for the FcRn (64–66).

The interspecies specificity of the binding of IgGs to FcRn has revealed the extreme selectivity of human FcRn for human IgGs. This explains the poor half-life in the human circulation of the first therapeutic IgGs of mouse origin. In stark contrast, the murine FcRn reacts with a high affinity to murine, human, and bovine IgGs. In particular, the affinity of the murine FcRn for human IgGs is much higher than that of the human FcRn (67, 68). Such an interspecies binding disparity was also demonstrated in the case of albumin, another FcRn ligand (69). The human FcRn has a greater affinity for murine albumin than for the human molecule. Conversely, the mouse FcRn binds murine albumin with a high affinity and also binds human albumin (70, 71). Such considerations are very important for the preclinical validation of therapeutic monoclonal antibodies and molecules that exploit the Fc- or albumin-fusion technologies and, for example, justify the use of human Fcγ1 fragments in the design of chimeric molecules.

**Figure 2:**
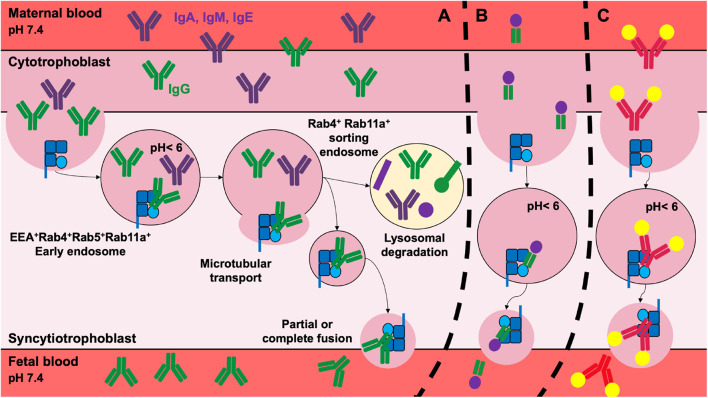
Transplacental delivery of maternal IgG and its therapeutic implications. **(A)** In the human, the transplacental delivery of maternal IgG starts during the second trimester of pregnancy. IgG cross the cytotrophoblast and syncytiotrophoblast cell layers to reach the fetal circulation. IgG transfer involves non-specific fluid phase internalization. IgG then colocalize with the FcRn in early endosomes where the acidic environment promotes FcRn/IgG interactions. Mature sorting endosomes transport FcRn/IgG complexes away from lysosomes, rescuing them from lysosomal degradation. IgG is released from FcRn into fetal blood by the partial or complete fusion of the endosome with the plasma membrane. After the dissociation of the IgG/FcRn complexes, FcRn returns to its original position. The transplacental delivery of Fcγ-fused proteins **(B)** such as FVIII-Fc or PPI-Fc, or of immune complexes **(C)** was validated for therapy in preclinical models in order to shape the fetal immune system. For simplicity, immune complexes are depicted as single IgG bound to two antigens.

The transcytosis of maternal IgG starts with the non-specific fluid phase internalization by intestinal or placenta epithelial or endothelial cells (5, 37, 72). Following their internalization, IgGs accumulate in Rab5^+^EEA^+^ early endosomes where they bind to FcRn upon pH acidification (73). The IgG/FcRn complexes are released in the intercellular space by partial or complete fusion of recycling endosomes with the plasma membrane (74, 75). Once at neutral pH, deprotonation of the histidines allows the dissociation of IgG from the FcRn (8) (Figure 2A).

The FcRn-dependent recycling pathway was also widely studied. As for IgGs transcytosis, IgG recycling begins with internalization by vascular endothelial cells and macrophages. IgGs accumulate in Rab5^+^EEA^+^ early endosomes where they colocalize with the FcRn. The binding of IgGs to the FcRn rescues them from the lysosomal degradation pathway. The matured Rab4^+^Rab11a^+^ sorting endosomes transport the complex away from lysosomes. In contrast, recombinant IgGs with a mutated His435, that do not bind FcRn, are routed to the lysosomes and are degraded (76–78).

## Functions of FcRn

### Role of The FcRn in IgG Transcytosis

As explained above, the FcRn was first identified for its role in the transfer of maternal IgGs to the baby during fetal life through the placenta and during breast-feeding through the digestive epithelium. During pregnancy in humans, maternal IgGs are detected in the umbilical cord from 8–10 weeks of gestation (GW8-10) (79). The concentration of maternal IgGs in the fetal circulation remains low until the second part of the second trimester (80) to reach 10% of maternal IgGs at GW22. It then increases to 50% at GW30 and exceeds the concentration in maternal blood at the end of the gestation (GW37-40) (81–84). It was proposed that the increased transfer at the end of gestation is due to the expansion of the exchange surface which grows from 5 m^2^ at GW28 to 11–12 m^2^ at the term (85). In humans, the majority of maternal IgGs are transferred across the placenta. In mice, a low but significant IgG transmission is detected at embryonic day 15 (E15) (86) that peaks at E17 (87). The majority of IgG is delivered after birth by ingestion of maternal milk. Antibodies in the colostrum and more generally in the maternal milk cross the intestinal barrier to reach the fetal circulation (46, 88). The rodent intestine is permeable to maternal IgGs until 20 days after birth (86–88).

The trans-epithelial and transplacental delivery of maternal IgGs plays an essential role for the protection of the newborn by providing passive immunity against a large array of pathogens. Passive immunity was observed in the 19th century during the measles epidemic, where babies from mothers who had survived were protected. The transfer of passive immunity was however first described by Paul Ehrlich in 1892, when he noticed that babies were protected against toxins only if the mothers were themselves resistant. Nowadays, vaccines against influenza, pertussis, diphtheria, meningococcus, measles, pneumonia and hepatitis are currently administrated to pregnant women to foster the development of protective IgGs that are then transferred to the fetus (89–95). The efficiency and duration of the transferred passive immunity however depends on the antigenic specificity of the IgG (91, 92, 96, 97).

### Role of The FcRn in The Recycling of Circulating IgG

Most plasma proteins and immunoglobulins have a short half-life (1-2 days) in the circulation. In contrast, IgGs present a half-life of 23 days in humans (98) and 7 days in mice (99). In the 60's, Brambell et al. proposed that IgG catabolism is regulated by the same receptor involved in IgG transfer: the FcRn (3, 100). This was formally demonstrated in models of ß-2-microglobulin deficient mice (26, 101–103) as well as in FcRn-deficient mice (94, 95) where IgG half-life was systematically reduced. Conversely, it was restored to normal in transgenic mice expressing the human FcRn (104). As described in the case of IgG transplacental or trans-epithelial delivery, IgG recycling involves fluid phase internalization by vascular endothelial cells and macrophages (31, 76, 105, 106) and binding by the IgG CH2 and CH3 domains to the FcRn in early endosomes (54, 98, 107, 108). The binding to the FcRn protects IgGs from lysosomal degradation and fosters their recycling to the circulation (37, 73, 77, 78). The FcRn-dependent recycling pathway of IgG is saturable and unbound IgGs accumulate in the lysosomes where they are degraded (76, 77, 103).

### Role of The FcRn in Antigen Capture and Presentation

The FcRn is expressed by a large variety of immune cells (109). Because of its structure homology with MHC class I molecules, FcRn was initially proposed to present endocytosed antigens. It was however demonstrated that the peptide-binding groove is occluded in the FcRn molecule (21). The FcRn is nevertheless indirectly implicated in antigen uptake and presentation (109). For instance, the expression of the FcRn on neutrophils was associated with the phagocytosis of IgG1-opsonized bacteria (42). Because the FcRn does not bind IgGs at neutral pH, it was proposed that immune complexes are captured and endocytosed by other FcR receptors. FcRn binding occurs in a second step once the pH acidifies; it allows sorting of the immune complexes to loading compartments and promotes antigen presentation as well as cross-presentation (109–111). The FcRn also transports IgGs from the intestinal basolateral side to the intestinal lumen where they form immune complexes with their cognate antigens. The immune complexes are then transported through the epithelium of the lamina propria where they are internalized by antigen presenting cells (i.e., dendritic cells) and presented to T cells (112–114).

### Role of The FcRn in The Recycling of Circulating Albumin

Albumin is the most abundant protein in plasma. It is involved in the transport of endogenous and exogenous molecules as well as in the maintenance of osmotic pressure (115). It is produced in high quantities and rapidly secreted by the liver (116) and is found in secretions such as tears, saliva, sweat and maternal milk. Albumin is characterized by an extended half-life in blood: it persists for 19 or 2-3 days in the human and mouse circulation, respectively (69, 117, 118). Such a long half-life is also mediated by binding to the FcRn. IgG and albumin bind FcRn at non-overlapping sites, without cooperation or competition. The interaction between albumin and FcRn is hydrophobic, depends on acidic pH and presents a 1:1 stoichiometry (119).

## Physiological Relevance of The Transfer of Maternal IgG

The following chapter summarizes the timing of the development of the fetal immune system in humans and mice. Notably, the establishment of the adaptive immune system and the generation of T and B lymphocytes expressing rearranged T-cell and B-cell receptors, respectively, at their surface is concomitant to the transplacental delivery of maternal IgGs, thus creating a time window when maternal IgGs, that represent the last step of the expression of the maternal immune system, have the opportunity to impact the developing adaptive immune repertoires of the fetus.

### Ontogeny of The Human Immune System During Fetal Life

**Figure 3:**
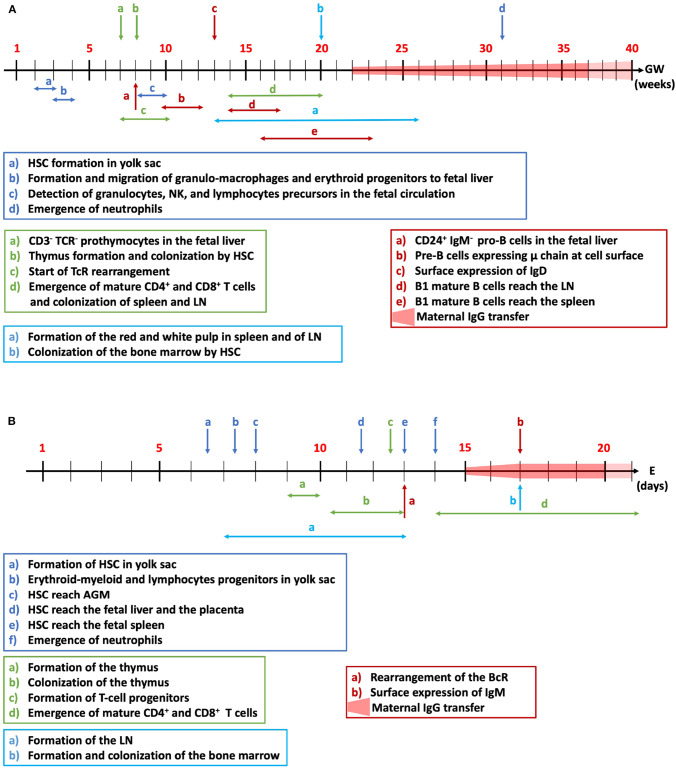
Fetal development of the immune system. The time-dependent ontogeny of the human **(A)** and mouse **(B)** immune systems is summarized for innate immune cells (dark blue), adaptive T (green) and B cells (red) and colonization of the lymph nodes and bone marrow (light blue). HSC, hematopoietic stem cells; NK, natural killer cells; TcR, T-cell receptor; LN, lymph nodes; BcR, B-cell receptor; AGM, aorta-gonad-mesonephros; GW, gestational weeks in the human; E, embryonic days in mice.

The development of the immune system starts after 2-3 weeks of fetal development with the initiation of hematopoiesis and generation of pluripotent and self-renewing hematopoietic stem cells (HSC) (Figure 3A) (120). In all mammals, hematopoiesis first occurs in the mesoderm of the yolk sac, and the extraembryonic mesenchymal tissue (121). Cells of the innate immune system are the first to emerge. Erythroid and granulo-macrophage multipotent progenitors, which give rise to megakaryocytes and myeloid cells, are detected from the gestational week (GW) 3 to 4. Dendritic cell-like cells are found in the yolk sac and the mesenchyme at GW4-6. From GW4, these progenitors are released in the circulation and reach the fetal liver, which becomes the major hematopoietic site until birth, when the bone marrow takes over (121). With respect to secondary lymphoid organs, the different subunits of the spleen form during GW13-28, and the red and white pulps are visible at the end of the second trimester (122). The development of lymph nodes occurs at the same period. The involvement of the spleen and lymph nodes (LN) in hematopoiesis, together with that of the fetal liver, ceases at birth (123). Between GW8-10, granulocytes, NK cells and lymphocyte precursors are detected in the fetal circulation (124). The GW12-19 fetal blood already contains high levels of erythroid, monocytic and granulocytic progenitors. Neutrophils are the last type of innate immune cells to be produced (GW31).

CD7^+^CD45^+^ pro-thymocytes with an intracytoplasmic CD3^+^ are detected in fetal liver from GW7. CD3 is not expressed at the surface of thymocytes until GW10 when the cells become less proliferative (125). T-cell receptor (TcR) rearrangement starts from GW6-9.5 and is first detected in the fetal liver before the thymus takes over (126). The colonization of the thymus by HSC starts at GW8 and the thymus organogenesis is complete at GW20 (127). Mature CD4 and CD8 single-positive T lymphocytes leave the thymus for the periphery and reach the spleen and LN from GW14 onwards (123).

Pro-B cells, which are characterized by the expression of CD24 and the absence of expression of IgM, are detected in the fetal liver at GW8. Pre-B cells, that emerge from the pro-B cell pool, express the immunoglobulin μ chain in the cytoplasm from GW8 onwards, and at the cell surface at GW10-12 (128). B-cell receptor (BcR) expression is necessary for B cell proliferation and migration to the periphery (129). IgD surface expression is detectable from GW13 and surface IgM levels are maximal around GW7-18. Immature B cells are released in the circulation and reach the LN at GW14-17 and the spleen at GW16-23 where they become mature B cells (123). Pre-B cell quantities decrease from GW13-23 in the fetal omentum (130). Despite the early burst of Ig production during fetal life, newborns have low quantities of IgM, IgA and IgE. The neonatal immune system responds to antigens mainly by producing IgM with low affinities (131). At birth, the majority of innate and adaptive cells are immature (132) but the immune system is functional and complete. The exposure to external antigens after birth promotes the adaptation and expansion of the immune system (133, 134).

### Ontogeny of The Mouse Immune System During Fetal Life

The development of the immune system in rodents involves, as in humans, the differentiation of pluripotent HSCs into myeloid or lymphoid lineage progenitors (135). In mice, hematopoiesis starts at embryonic day (E) 6.5 (Figure 3B). The first type of HSC, the erythro-myeloid, and lymphoid progenitor cells are formed in the yolk sac at E7.25 (136) and macrophages and monocytes appear at E9 (137). At E10, HSCs are detected in the aorta-gonad-mesonephros (AGM) (138). Fetal circulation is established at E8.5, allowing HSCs to leave the AGM and to reach the fetal liver and the placenta, the two main reservoirs of HSCs at mid-gestation (E11.5) (139). The development of LN starts between E7 and E13 depending on their localization (140). LN are rapidly colonized by T cells and the first LN follicles are formed 1 week after birth (141). At E13, HSC and lineage-restricted progenitors reach the fetal spleen (123). The lymphatic network is established at E15.5 (142). Formation of the bone marrow is one of the last stages of mice development (E17). At E17.5, HSCs and lineage-specific progenitors leave the liver to colonize the bone marrow (143) where they remain until adulthood. Bone marrow HSCs form the first reserve of stem cells for post-natal life. After E18, the bone marrow assumes the maintenance of the HSC pool and the development of hematopoietic cells.

Neutrophils are detected in the circulation for the first time at E14 at very low numbers (<2%) and reach 20% at E18 (144). Neutrophils and monocytes are, at the fetal and newborn stages, the first line of defense against infection. At E13–15.5, hematopoiesis switches from the liver to the thymus for T cells and the spleen for B cells. The thymus anlage is detected from E9-10 (123) and its colonization by lymphoid progenitor cells occurs between E10.5 and E13 (145). T-cell progenitors are first synthesized from lymphocyte progenitors at E12.5. Thymocytes first express the TcR and then undergo positive and negative selection, to eliminate auto-reactive clones. In the late gestational period (E14-21), simple positive CD4 or CD8 T cells are produced. The bone marrow is the main organ where B-cell lymphopoiesis takes place (146). The rearrangement of the genes encoding the B-cell receptor initiates by E13 and IgM^+^ B cells are detected at E17 (147).

### Shaping of Adaptive Immune Repertoires by Maternal IgG

Transplacentally delivered maternal IgGs are important for the protection of newborns from bacterial or viral infections. Importantly, the transfer of maternal antigen-specific IgGs influences antigen-specific immune responses later in the life by altering both the repertoires of T and B lymphocytes in the progeny. Seminal work by Faure et al. demonstrated that the transfer of κ light chain-specific maternal IgGs alters the repertoires of κ light chain-specific T cells and confers a transient state of tolerance toward peptides derived from the constant region of the κ light chain (148). This was demonstrated by following κ light chain constant region (Cκ)-specific CD4^+^ T cells in κ light chain knock-out (κ^−/−^) mice born to κ^+/−^ mothers. Hence, the transfer of maternal IgGs from mothers bearing a κ light chain to κ light chain-deficient fetuses altered in an antigen-dependent manner the repertoires of T lymphocytes (148).

In the B cell compartment, early idiotypic manipulations via maternal immunization with antigens or monoclonal IgGs, or after treatment of newborns with anti-idiotypic IgGs, were shown to induce profound states of tolerance toward the particular idiotype (149, 150). In such systems, the suppression of antibody responses was always reversible. Its recovery was associated with the expression of the same (151) or different idiotypic repertoires (152–154). For instance, the transfer of maternal anti-idiotypic IgGs directed against anti-phosphorylcholine (PC) antibodies skewed the repertoires of PC-specific B lymphocytes after immunization of the offspring with PC later in life (155).

Another example of the importance of normal IgGs in shaping immune repertoires is provided by studies on intravenous immunoglobulins for therapeutic use (IVIG). Exploration of the mechanisms of action of IVIG led to identification of various F(ab')_2_-dependent mechanisms. Through anti-idiotypic interaction, IVIG neutralizes pathogenic autoantibodies and shapes the repertoire of auto-IgG-producing B-cell clones (156). IVIG reciprocally regulates pathogenic Th1/Th17 cells and immune-protective regulatory T cells by F(ab')_2_-dependent process (157, 158). While both F(ab')_2_- and Fc-dependent regulation of dendritic cells and macrophages by IVIG have been reported (159–162), F(ab')_2_ fragments of IVIG regulate the functions and repertoires of granulocytes like eosinophils, basophils and neutrophils (163, 164). In line with these functions, auto-antibodies to diverse self-molecules have been identified and isolated from IVIG including HLA class I, CD40, adhesion molecules, CD4, CD5, Siglecs, IgE, and Fas/CD95 (156).

## Pathological Implications of The Transplacental Delivery of Maternal IgG

The transfer of maternal IgGs to the fetus may have pathological repercussions when the mothers present with autoimmune disorders caused by self-reactive IgG. In such situations, the FcRn plays a dual role, increasing disease severity in the mothers by controlling the concentration of circulating pathogenic IgGs, and mediating the transmission of pathogenic IgGs to the fetus thereby inducing disease manifestations. A typical example is the transfer of the Sjögren's syndrome upon transplacental delivery of maternal autoantibodies directed to the nuclear proteins Ro/SSA and La/SSB (165). The Sjögren's syndrome affects ~1/10,000 adults with a majority of women (90%) (166, 167). Anti-SSA/Ro and SSB/La IgG target the Ro/La ribonucleoprotein complex constituted by two Ro protein isoforms (52 kDa and 60 kDa) and the La protein (48 kDa). Ro52 is involved in the regulation of proliferation and cell death (168) and in the regulation of interferon regulator factor-mediated immune responses (169, 170), while Ro60 is implicated in the control of RNA integrity (171). The translocation of these antigens at the surface of salivary gland cells allows their targeting by autoantibodies, leading to dysfunction of the exocrine glands, lymphocytic infiltrates in the salivary gland and parotid gland enlargement (172, 173).

In ~2% of babies from Sjögren's syndrome-affected mothers (174), the transfer of maternal anti-SSA/Ro and SSB/La is responsible for the development of neonatal lupus erythematosus (NLE) leading to the development of rashes, liver damage, neuropsychiatric impairment (175) or congenital heart block (CHB). CHB presents with a mortality rate of 18% and requires implantation of a pacemaker in 70% of the cases (176, 177). Mothers who give birth to CHB-affected children possess anti-Ro/anti-LA IgG and may be either asymptomatic or present with systemic lupus erythematosus, Sjögren's syndrome or undifferentiated autoimmune diseases (178, 179). In the case of anti-SSA/Ro and SSB/La IgG-mediated CHB, the autoantibodies either target the autoantigen that has translocated on the cell surface of apoptotic cardiomyocyte (180, 181) and/or cross-react with L-type calcium channels (LTCCs) present on the cardiomyocyte surface (182). The interaction between autoantibodies and autoantigens leads to immune complex deposition, inflammation, disruption of calcium homeostasis and calcification, heart fibrosis and signal conduction blockade in the atrioventricular node (183, 184).

The hemolytic disease of the fetus and newborn (HDFN) is another example of the contribution of maternal IgGs to the development of fetal pathologies (185). The maternal IgGs are directed against Rhesus (Rh) antigens (RhD, RhC, RhE, K, M, …) expressed by fetal erythroid cells, and are either self-reactive or have developed against fetal antigens during a previous pregnancy. The ensuing destruction of red blood cells induces anemia which in the worst cases results in perinatal mortality and morbidity (186, 187). The prevalence of HDFN caused by anti-Rh antibodies others than anti-RhD is 1 in 500 pregnancies (185).

IgG specific for platelet membrane glycoproteins may also be transferred from the mothers to fetuses. Thus, anti-platelet autoreactive IgGs develop in 1/500 pregnancy leading to a disease called autoimmune thrombocytopenia (188). Autoimmune thrombocytopenia is characterized by a reduced quantity of platelets and the development of mucocutaneous bleeding. Alternatively, 1 in 2,000 mothers develops alloantibodies directed against paternally derived platelet antigens. Transplacentally delivered maternal anti-platelet autoimmune or alloimmune IgGs target fetal platelets causing the development of fetal thrombocytopenia which, in 1 or 20% of the cases, respectively, is severe and causes intracranial hemorrhages (189, 190).

More anecdotical, the presence of autoreactive IgGs against neuronal and glial proteins or of IgGs induced by maternal infections has been associated with autism spectrum disorders (191), although available epidemiologic data are too scarce to confirm any association.

In the mice, the FcRn-mediated transfer of maternal IgE in the form of IgE/IgG anti-IgE complexes has been associated with the development of allergic disease (192, 193).

## Therapeutic Value of FcRn-Mediated Delivery

### Increasing the Half-Life of Biological Therapeutics

The capacity of the FcRn to extend the pharmacokinetics of therapeutic molecules has been exploited in several instances. To this end, therapeutic molecules are fused with the Fc fragment of human IgG, human albumin or an albumin-binding domain. The first Fc-fused molecule accepted by the FDA was a chimera between the TNF receptor and the human Fcγ and is used for the treatment of rheumatoid arthritis (194). Nowadays, several Fc-fused molecules are approved for clinical use, including drugs for the treatment of immune thrombocytopenic purpura (195), asthma, psoriasis, etc. [reviewed in Rath et al. (196)]. Notably, the Fc fusion technology has been used in the field of hemophilia. The Fc fusion of coagulation factor XI and of pro-coagulant factor VIII (FVIII), that have short intrinsic half-lives, was shown to increase the half-life of the molecules in the patients, thus, allowing the reduction of injection frequency (68, 197). More recently, modifications of the CH2 and CH3 domains of the human Fcγ by mutagenesis have allowed an increase in the affinity for the FcRn and thus further extend the pharmacokinetic of Fc-fused products (198–200). Of note, targeting albumin (201–204) or using albumin-fusion technology is also used in the case of coagulation factor IX for the treatment of hemophilia B (205), as well as for biotherapeutics for the treatment of diabetes (201, 206), cancer (202, 204) or rheumatoid arthritis (207).

### Saturation of The IgG Recycling Pathway

As explained earlier, the FcRn-dependent recycling pathway is saturable. This property has been exploited as a strategy to eliminate endogenous pathogenic IgGs. Historically the recycling pathway was saturated with IVIG injected in large amounts. IVIG compete with endogenous IgGs for the binding to the FcRn, thus promoting their routing to the lysosomal degradation pathway and lowering their levels in the circulation (208, 209). Nevertheless, owing to the cumbersome procedures as well as cost and possible side effects associated with IVIG treatment, alternative therapies are being developed. Novel molecules, referred to as “antibodies that enhance IgG degradation” or “Abdegs” (210), that bind to the FcRn with a higher affinity than IgG and in a pH-independent manner, have recently been generated. Moreover, FcRn-blocking monoclonal antibodies, such as Rozanolixizumab (211), SYNT001 (212), M281 (213) and Efgartigimod (214) are currently in phase 2 or 3 clinical trials (NCT04200456, NCT03075878, NCT04119050, NCT04225156). These molecules hold promise for the treatment of IgG-mediated diseases such as systemic lupus erythematosus, myasthenia gravis or immune thrombocytopenic purpura (210, 215).

### Shaping of The Immune System in The Offspring

The capacity of maternal IgGs to cross the placenta during pregnancy or the epithelial barrier during breastfeeding in an active FcRn-dependent manner can be exploited to educate the immune system of the offspring and confer protection in several human pathologies such as asthma, type-1 diabetes (T1D), hemophilia A (Figures 2B,C). Allergic asthma is one of the most represented allergic diseases with, according to the WHO, 235 million people affected (216). Allergic diseases have an increased prevalence, particularly in developed countries owing to changes in lifestyle (217) and environmental exposure during early life (218). Asthma develops following the polarization of CD4^+^ T cells toward a Th2 subtype, upon activation by usually innocuous inhaled or ingested allergens. The secretion of IL-4 by Th2 cells induces the differentiation of B cells into plasma cells, which secrete allergen-specific IgE. IgE-allergen immune complexes then interact with mast cells through the FcεR, leading to degranulation and release of vasoactive amines (219). Asthma is characterized by a chronic inflammation of the lungs and mucus accumulation, causing respiratory difficulties (220, 221). During pregnancy, allergens inhaled or ingested by the mother shape the immune system of the fetus (222). Indeed, allergens contained in mothers' diets were proposed to cross the placenta and to be present in maternal milk (223). As described in the case of passive protection conferred by maternal IgG against infectious, breastfeeding protects children against the development of asthma. Such a protection implicates the transmission of the antigen in the form of IgG immune complexes, in a FcRn-dependent manner leading to the induction of active immune tolerance (224, 225). Importantly, for tolerance to be induced, mothers have to be exposed to allergens during the breastfeeding period (226–228). In the mouse, breastfeeding by mothers sensitized to ovalbumin (OVA), used as a model allergen, promotes a higher induction of tolerance in the progeny than breastfeeding by non-sensitized mothers. Transmission of the allergen from the mothers to the offspring induces OVA-specific regulatory T cells (Tregs), which proliferate and suppress Th2 responses in an allergen-specific manner (229, 230). Depletion of allergen-specific Tregs abolished protection in the pups. Importantly, the induction of OVA-specific Tregs was dependent on the transfer to babies through the FcRn of allergen-IgG immune complexes contained in the breast milk, as shown by the fact that FcRn-deficient mice breastfed by exposed mothers were not protected from the development of asthma (229). Interestingly, the protection against allergens conferred by breastfeeding is sustained beyond the elimination of maternal IgG from the offspring circulation (229). Recent studies show that following FcRn-mediated delivery of OVA-IgG immune complexes, the allergens are internalized by neonatal conventional DC (cDC) (230). While antigen-IgG immune complexes may first be transferred through the placenta, transfer of the allergen through maternal milk may be necessary to get optimal protection. Whether the preventive administration to human of allergen-containing IgG immune complexes may reduce the incidence of asthma in individuals at risk remains to be established.

Education of the fetus' or newborn's immune system by antigens delivered by maternal IgGs may occur spontaneously as explained above. The intentional transplacental delivery of disease-relevant antigens exploiting the FcRn as a Trojan horse from the mothers' circulation to the fetus' was recently validated in two experimental models of human diseases: T1D and alloimmunization to therapeutic FVIII in hemophilia A. Hemophilia A is a rare X-linked hemorrhagic disorder characterized by the lack of functional pro-coagulant FVIII. Bleedings are treated or prevented by the intravenous administration of therapeutic FVIII. The main complication in FVIII replacement therapy is the development of a specific IgG-mediated neutralizing anti-FVIII immune response (231). Several interventional strategies have been attempted in FVIII-deficient mice, an animal model of severe hemophilia A, in order to induce FVIII-specific immune tolerance (232). Among these, we demonstrated that the injection to pregnant FVIII-KO mice of the immunodominant A2 and C2 domains of FVIII fused to mouse Fcγ1 allows the transplacental delivery of A2Fc and C2Fc. The A2Fc and C2Fc were captured by SIRPα^+^ migratory conventional DCs (cDCs) and reached the fetal thymus where they induced antigen-specific natural Tregs. The immune response to exogenous FVIII was drastically reduced following replacement therapy in offspring from A2Fc/C2Fc treated mothers as compared to offspring from control mothers (233).

T1D is a multifactorial autoimmune disease characterized by the destruction of the insulin-producing ß cells of the pancreas. The incidence of T1D is increasing with an estimate of 420 million individuals affected world-wide (234). Destruction of ß cells by autoreactive T cells causes a deficiency in insulin leading to glucose metabolism impairment. People with T1D may develop blindness, heart attack, kidney failure, … Insulin is one of many self-antigens targeted by pathogenic T cells in T1D (235). Using G9Cα^−/−^.NOD mice that express a transgenic TcR derived from the insulin-reactive G9C8 CD8 T-cell clone and using NOD mice, a model of spontaneous T1D development, Culina et al. were able to delay the onset and reduce the incidence of T1D in offspring from mothers injected with a preproinsulin (PPI)-Fcγ1 fusion protein (236). As shown in the case of FVIII-Fc fusion proteins, PPI-Fc injected during pregnancy was delivered through the syncytiotrophoblast to the fetuses and was captured by SIRPα^+^ migratory cDCs. Unexpectedly, the presence of the antigen led to an increase in the recruitment of CD8^+^ T cells at the periphery, the cells were however less cytotoxic. The low affinity of the TcR from G9C8 CD8^+^ T cells for its target peptide allowed the induction of specific Tregs.

The capacity of FcRn to transfer maternal IgGs to the baby's circulation has also been exploited with the mere objective of correcting congenital deficiencies in essential enzymatic activities, referred to as lysosomal storage diseases. Lysosomal storage diseases represent a large panel of pathologies characterized by deficiencies in lysosomal enzymes that cause the accumulation of non-digested proteins in the lysosomes of various organs. The affected individuals develop variable morbidities ranging from severe physical impairment to death. Mucopolysaccharidoses (MPS) are members of lysosomal storage diseases and are caused by deficiencies in enzymes involved in the degradation of glycosaminoglycans in the lysosomes. In particular, MPS VII is caused by a deficiency in B-glucuronidase enzyme (GUS) (237). MPS are currently treated by intravenous administration of the lacking enzymes (238–240). Enzyme replacement therapy (ERT) is however hampered by the rapid clearance of the therapeutic enzymes, and by the fact that large amounts of enzymes are required to achieve a modest clearance of the non-digested lysosomal proteins (241). Importantly, ERT is also complicated by the development of neutralizing antibodies. In 2008, Grubb et al. injected pregnant MPS mice with a Fc-fused GUS enzyme. The GUS-Fc chimeric protein was transplacentally delivered to the fetuses in a FcRn-dependent manner (242, 243). After reaching the fetal circulation, the GUS-Fc distributed to brain, liver, spleen, heart, kidneys, lungs and eyes where it was as active as the native enzyme and resolved protein accumulation. Whether the strategy was able to induce tolerance to GUS-Fc was not reported, however.

## Conclusions

The combination of a better understanding of the mechanisms underlying the transplacental delivery of maternal IgGs with the advent of the Fc-fusion technology is opening a novel therapeutic field. Indeed, taking advantage of the FcRn-dependent materno-fetal interface should lead in the near future to new therapies to confer immune tolerance to antigenic targets of pathogenic immune responses. Despite the promise hold by this strategy, several challenges remain. While Fc-mediated transfer of antigen induces long-lasting (i.e., tested until 7-8 weeks of age) immune tolerance in preclinical mouse models, there is no data as yet to suggest that the same is true in primates, and it is adventurous to anticipate the long-term effects on the immune system of the offspring, notably in organisms with longer life expectancies. In addition, questions related to the dose of Fc-fused antigens to be injected to the pregnant mothers and optimal time-window for administration remain to be addressed.

Another aspect relates to the identification of the patients who will benefit from such preventive treatments. For instance, in the case of hemophilia A, 5–30% of the patients develop neutralizing anti-FVIII IgGs (244). Several risk factors have been identified as increasing the probability for a patient to develop allo-antibodies to therapeutic FVIII (i.e., disease severity, polymorphisms in immune genes, ability to control inflammatory and immune responses) (244). Yet, it is nowadays impossible to discriminate with certainty patients who will develop neutralizing anti-FVIII IgGs from those who will not. Among patients with the highest risk, the odds would be to treat three patients to prevent the pathogenic immune response that should develop in one of them. The situation is obviously less favorable in the case of diseases, the onset of which is more complicated to predict than alloimmunization to therapeutic FVIII in hemophilia A, such as T1D, or for which the target antigen is not known.

## Author Contributions

AM, JD, SK, JB, and SL-D: prepared outline of the review. AM, SD, IP, VD, ML, JB, and SL-D: wrote the review. AM and SL-D: draw figures.

## Conflict of Interest

The authors declare that the research was conducted in the absence of any commercial or financial relationships that could be construed as a potential conflict of interest. The reviewer MP declared a past co-authorship with one of the authors SL-D to the handling Editor.
